# Notes and News

**Published:** 1903-07-18

**Authors:** 


### Notes and News.

The King paid a surprise visit to the Princess Alice Memorial Hospital on Monday morning before leaving Eastbourne. The King as Prince of Wales opened the hospital 20 years ago, and has since presented an operation-table to it. An inscription in the visitors' book on Monday testifies to the favourable impression made on the King on the occasion of his early visit.

The Prince of Wales has consented to become a member of the British Medical Association.

The Prince of Wales, as Grand Prior of the Order of St. John of Jerusalem, has sanctioned the proposal that a silver challenge shield should be offered for competition amongst the employées of the Indian railways, as is the case in England and Wales.

The Royal Hospital for Children and Women, Waterloo Bridge Road, needs, we are informed, £50,000 for rebuilding, not the smaller sum of £5,000 which we mentioned in error last week. At a meeting of the ladies' executive committee in connection with the rebuilding of the institution, at which the Duchess of Albany presided, on Monday, it was decided that the ceremony of laying the foundation stone should take place on October 22nd.

The Belgrave Hospital for Children has been closed for some time. Now new premises have been erected in the Clapham Road, and the hospital is to be formally opened by her Royal Highness Princess Henry of Battenberg on Monday, July 20th, at 4.30 p.m. At present the building erected provides three wards, in addition to the babies' ward, giving altogether about 35 cots, with operating theatre and extensive out-patients' department, with physicians' and surgeons' consulting-rooms, isolated wards, etc., all on the most approved modern system. To start and work the hospital funds are urgently needed, and an anonymous donor has offered £2,000 if a like sum can be subscribed in sums of £25 each, but smaller donations will be thankfully received and acknowledged by the secretary.

A considerable increase of plague is reported from Port Said.

Lord Balfour received on Wednesday a deputation in support of the provision of a Government vaccine establishment.

The Metropolitan Hospital Sunday Fund now exceeds £48,000, irrespective of the promised contribution from Mr. George Herring. It is now expected that the collection will prove to be an excellent one this year.

The quiet little town of Shifnal, Salop, was stirred to unusual excitement on Saturday, the 27th ult., when the first Hospital Saturday was inaugurated on behalf of the Shrewsbury Infirmary and the Shifnal Cottage Hospital, a sum of over £60 being realised.

Another enemy to the mosquito is reported to have been discovered by Dr. Stiles, of the Washington Public Health Service, in the form of a parasite. This parasite is said to be deadly to mosquitos only, and Dr. Stiles is experimenting on the propagation of it, with a view to its distribution in mosquito-infested regions.

Mr. J. Tweedy, F.R.C.S., Professor of Ophthalmic Medicine and Surgery at University College, has been elected President of the Royal College of Surgeons of England, and Mr. G. H. Makins, L.R.C.S., and Mr. C. T. Dent, F.R.C.S., have been elected, and Mr. T. Butlin, F.R.C.S., re-elected, to the Council of the College.

At the annual meeting of the Association for Promoting the Welfare of the Feeble-minded a resolution was passed advocating a travelling commission to make a thorough investigation of the needs of the feeble-minded amongst the community. Dr. Shuttleworth stated that about one child out of every hundred required special care of some sort.

The Sanitary Institute, which has held a satisfactory congress at Bradford during the past week, closed its proceedings on Friday by passing a resolution in favour of the appointment of a Minister of Public Health, with a seat in the Cabinet, and also the separation and enlargement of the medical department of the Local Government Board into a Health Ministry.

The following is the prize list (summer session) of the Royal College of Surgeons in Ireland:—Mayne Scholarship—£15, R. W. Burkitt. Carmichael Scholarship—£15, I. Allaun. Gold and Silver Medals in Operative Surgery—Gold: A. N. Crawford. Silver: F. J. Cairns. Honour Certificate: C. A. Cusack. Practical Histology—W. St. Leger Moorhead, first prize (£2) and medal; S. H. Massey, second prize (£1) and certificate. Practical Chemistry—D. P. Clement, first prize (£2) and medal; H. C. Carden, second prize (£1) and certificate. Public Health and Forensic Medicine—Miss C. O'Meara, first prize (£2) and medal; T. W. Browne, second prize (£1) and certificate. Materia Medica—R. M. Bronte, first prize (£2) and medal; A. J. Faulkner, second prize (£1) and certificate. Biology—F. M. Hewson, first prize (£2) and medal; W. A. Ryan, second prize (£1) and certificate.

